# Supplementary material for: Identification and functional analysis of non-coding regulatory small RNA FenSr3 in Bacillus amyloliquefaciens LPB-18
Source: PeerJ. 2023 May 15;11:e15236. doi: 10.7717/peerj.15236 (PMC10194069; doi:10.7717/peerj.15236)
Supplement: Supplemental Information 4 [file peerj-11-15236-s004.zip › KO/CK-vs-T1_map/map04122.html]

KEGG PATHWAY: Sulfur relay system - Reference pathway


|  |  |
| --- | --- |
| **Sulfur relay system - Reference pathway** |  |

[
Pathway menu
| Organism menu
| Pathway entry
| Show description
| User data mapping
]

Ubiquitin and ubiquitin-like proteins (Ubls) are signalling messengers that control many cellular functions, such as cell proliferation, apoptosis, and DNA repair. It is suggested that Ub-protein modification evolved from prokaryotic sulfurtransfer systems. Molybdenum cofactor (Moco) and thiamin are sulfur-containing cofactors whose biosynthesis includes a key sulfur transfer step that uses unique sulfur carrier proteins, MoaD and ThiS. Ubiquitin, MoaD, and ThiS are all structurally related proteins whose C-termini are activated through adenylation by homologous E1-like enzymes. s2T biosynthesis may share similar chemistry with Moco and thiamin synthesis. In Saccharomyces cerevisiae, Urm1 and Uba4 function as part of a ubl protein conjugation system, though they have sequence homology to bacterial sulfur-transfer enzymes and the ability to function in sulfur transfer.

|  |  |  |
| --- | --- | --- |
| Reference pathway | 184% 150% 122% 100% 82% 67% 55% | 图片下载 |
